# Tolerability of Palmitoylethanolamide in a Pediatric Population Suffering from Migraine: A Pilot Study

**DOI:** 10.1155/2020/3938640

**Published:** 2020-04-24

**Authors:** Laura Papetti, Giorgia Sforza, Giulia Tullo, Pierfrancesco Alaimo di Loro, Romina Moavero, Fabiana Ursitti, Michela Ada Noris Ferilli, Samuela Tarantino, Federico Vigevano, Massimiliano Valeriani

**Affiliations:** ^1^Headache Center, Department of Neuroscience, Bambino Gesù Children Hospital in Rome, Rome, Italy; ^2^Child Neurology Unit, Systems Medicine Department, Tor Vergata University Hospital of Rome, Rome, Italy; ^3^Department of Neurosciences, Mental Health, and Sensory Organs (NESMOS), Faculty of Medicine and Psychology, Sant'Andrea Hospital, Sapienza University, Rome, Italy; ^4^Department of Statistical Sciences, Sapienza University of Rome, Rome, Italy; ^5^Center for Sensory-Motor Interaction, Aalborg University, Denmark Neurology Unit, Aalborg, Denmark

## Abstract

**Background:**

Palmitoylethanolamide (PEA) is emerging as a new therapeutic approach in pain and inflammatory conditions, and it has been evaluated in studies on various painful diseases. The aim of this open-label study was to evaluate the efficacy of ultramicronized PEA (umPEA) in the prophylactic treatment of migraine.

**Methods:**

The study included 70 patients with mean age of 10.3 ± 2.7 (24.5% M and 75.5% F). All patients had a diagnosis of migraine without aura (ICHD 3 criteria) and received umPEA (600 mg/day orally) for three months. We compared the attack frequency (AF) and attack intensity at baseline and after three months. Patients were asked to classify the intensity of the attack with a value ranging from 1 to 3, where 1 means mild attack, 2 moderate, and 3 severe attack.

**Results:**

Nine patients discontinued treatment before the target time of 12 weeks. After 3 months of treatment with umPEA, the headache frequency was reduced by >50% per month in 63.9% patients. The number of monthly attacks at *T*_1_ decreased significantly compared with the baseline assessment (from 13.9 ± 7.5 SD of *T*_0_ to 6.5 ± 5.9 SD of *T*_1_; *p* < 0.001). The mean intensity of the attacks dropped from 1.67 ± 0.6 (*T*_0_) to 1.16 ± 0.5 (*T*_1_) (*p* < 0.001), and the percentage of patients with severe attacks decreased after treatment (from 8.2% to 1.6%; *p* < 0.05). The monthly assumptions of drugs for the attack reduced from 9.5 ± 4.4 to 4.9 ± 2.5 (*p* < 0.001). Only one patient developed mild side effects (nausea and floating).

**Conclusions:**

Our preliminary data show that umPEA administered for three month reduces pain intensity and the number of attacks per month in pediatric patients with migraine. Although the small number of patients and the lack of control group do not allow us to consider these initial results as definitely reliable, they encourage us to expand the sample.

## 1. Introduction

Migraine is a frequent disabling disorder in children and adolescents, with recent meta-analytic data estimating its prevalence at 7.7% in this age group [1, 2]. Episodic migraine (EM), in which headache attacks involve less than 15 days per month, affects approximately 5% of children up to the age of 12 years and 11% of adolescents [3]. Chronic migraine (CM) is very disabling and is characterized by 15 or more days per month with headache for at least three months. CM is not uncommon in the pediatric population affecting from 0.6% to 1.8% of children and adolescents [4–6]. Traditionally, pediatric migraine treatment includes both prophylactic therapy, aiming at reducing the severity and frequency of attacks and acute therapy to stop the attack pain.

Although amitriptyline, topiramate, and flunarizine have the most solid data supporting their use for migraine prophylaxis in children, a serious lack of controlled studies on the pharmacological treatment still remains [7, 8].

In absence of consistent, high-quality efficacy data for the use of pharmacologic preventive migraine interventions targeted at children and adolescents, another modality worth to be explored consists in the use of nonpharmacologic pill-based interventions including nutraceuticals. Nutraceuticals may be offered to parents who are reluctant to start their child on a daily medication [8].

Despite nutraceuticals are largely used for prophylactic treatment of children's headache, there is a lack of official guideline. Even the few available studies provide limited evidence of nutraceutical efficacy [9]. The most frequently used nutraceuticals for headache's prevention in children and adolescents are magnesium, coenzyme Q10 (Cq10), riboflavin, butterbur, melatonin, and preparations of feverfew [9–11].

Palmitoylethanolamide (PEA) is an endogenous fatty acid amide widely distributed in different tissues, including nervous tissues. This compound is naturally produced in many plant and animal food sources as well as in cells and tissues of mammals and endowed with important neuroprotective, anti-inflammatory, and analgesic actions. Several efforts have been made to identify the molecular mechanism of action of PEA and explain its multiple effects both in the central and the peripheral nervous system [12].

PEA has been reported to be effective in animal models of chronic pain and inflammation as well as in several clinical trials on various pain and inflammatory conditions. [12–16].

However, to date, only one study has been conducted to evaluate the role of PEA in migraine management in adults with a statistically significant and time-dependent pain relief [17].

The aim of this open-label study was to evaluate the safety and the efficacy of um-PEA in terms of reducing the frequency and severity of migraine attacks in pediatric patients.

## 2. Methods

We performed a prospective open-label study from January 2018 to January 2019 in patients admitted to the Headache Center of Bambino Gesù Children Hospital in Rome. The Hospital Ethics Committee approved the study protocol, and the parents of enrolled patients signed the written informed consent.

### 2.1. Subject Recruitment

Inclusion criteria included diagnosis of episodic migraine without aura according to ICHD 3 criteria [18]: age between 5 and 17 years and high frequency of the attacks (more than 4 attacks and less than 15 attacks for month). Exclusion criteria included the following: chronic migraine according to ICHD 3; concomitant history of medication overuse headache (MOH); treatment with other prophylaxis drugs, including nutraceuticals, in the three months prior to recruitment; progressive serious clinical conditions (cancer, chronic hepatitis, and human immunodeficiency virus); neuropsychiatric diseases (e.g., psychosis and depression, for the risk of low compliance), renal diseases (serum creatinine concentration more than 1.2 times the upper limit of the normal range according to the central laboratory reference values); and liver dysfunction (serum alanine or aspartate transaminase concentration more than 1.5 times the upper limit of normal range according to the central laboratory reference values).

In order to assess the efficacy of treatment, we compared the monthly attack frequency (AF) and attack intensity (AI) at baseline (*T*_0_) and after three months of therapy (*T*_1_). Patients were asked to classify AI with a value ranging from 1 to 3 where 1 means mild attack, 2 moderate, and 3 severe attack. The primary endpoints were the reduction of the frequency of the attacks more than 50% respect to the baseline and the reduction of pain intensity of at least one point.

### 2.2. Study Design

The study was conducted in three phases. A first (prescreening) visit was used to identify candidate patients based on the inclusion criteria. These patients gave written consent form to participate in the subsequent phases of the study. In a second phase, the enrolled patients carried out a one-month observation period where they reported on a diary the frequency and intensity of attacks and the number of intake of drugs for the attack. Baseline data were then extrapolated from the patient diary. Third, the patients began the treatment phase during which they took umPEA at doses of 600 mg/day divided in two doses.

During the treatment phase, they continued to record the data of frequency and intensity of the attacks and the number of intake of drugs for the attack in a diary. Only data from patients who completed a minimum 3-month therapy period were considered for posttreatment data collection (*T*_1_). Safety was assessed by monitoring the incidence of adverse drug reactions (ADRs) which were classified according to both severity and causality.

### 2.3. Statistical Analysis

Statistical analysis was conducted with SPSS software version 22.0. The differences between multiple means before and after treatment were assessed using *T*-student test and Wilcoxon signed-rank test. The linear mixed models (LMMs) were used to verify the effect of different parameters (age, sex, and duration of treatment) on the frequency and intensity of the attacks and assumption of drugs before and after treatment with PEA (age, sex, and duration of treatment). In particular, we adopted the generalized linear mixed model (GLMM) for evaluating the effect on frequency and assumptions of drugs for the attack (continuous variables) and cumulative linear mixed model (CLMM) for the intensity of the attacks (categorial variable). For both GLMM and CLMM, we assumed the Poisson distribution and the models intercepted through link log an individual female before the therapy as a reference subject. Logistic regression was used to verify a correlation between age and frequency of the attacks.

As for the dose of drug chosen, we referred to the minimum effective dosage reported by other works on chronic pain, equal to 600 mg/day. However, since the age group of the subjects studied is very wide (from 6 to 17 years) and this implies a variability of the wight of the subjects, we tried to find per kilo dose considered effective. We then calculated the average dosage per kilogram of body weight for patients who achieved a reduction in the number of attacks in *T*_1_ greater than 50%.

We therefore compared the results in three categories of subjects: the patients who carried out the therapy for at least 12 weeks (group A), the dropout patients (group B: less than 12 week, side effects or incorrect reports), and the total population (group A plus B).

The significant level of statistical result was established for *p* < 0.05.

## 3. Results

The study included 69 patients with mean age of 10.43 ± 2.8 years (range between 5.4 and 17.6 years old). The population included 26.1% male and 73.9% female. All patients had a diagnosis of episodic migraine without aura (ICHD 3 criteria). In all enrolled patients, the laboratory parameters were in the normal range, thus excluding systemic diseases. No enrolled patients were receiving any prophylactic treatment for migraine, but during the acute headache, nonsteroidal anti-inflammatory drugs (NSAIDs) like ibuprofen, paracetamol, diclofenac sodium, and ketorolac were used.

Sixty-one children received umPEA (600 mg/day orally) for three months, while eight patients left the study earlier. Main causes of dropout from the study included early discontinuation of treatment (5/8 patients), incorrect compilation of the diary (2/8) and reported side effects (1/8). For patients with incorrect diary and side effects, we considered *T*_1_, the time when the correct report of the attacks stopped or side effects were developed. The mean duration of patients treated for less than 12 weeks was 4 ± 1 weeks.

### 3.1. Effects on Frequency of the Attacks

We found that, after 12 weeks of treatment with umPEA, the headache attack frequency was reduced by >50% in 63.9% patients (vs 56.5% of group B).

**Figure 1:**
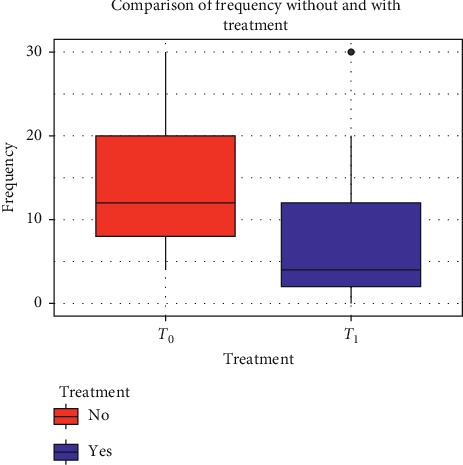
Comparison of frequency of migraine attacks before and after 12 weeks of treatment with umPEA.

The number of monthly attacks at *T*_1_ decreased significantly compared with the baseline assessment in the total subject (from 14.06 ± 7.8 SD of *T*_0_ to 7.2 ± 6.4 SD of *T*_1_; mean difference 6.85; *p* < 0.001) and in the group A (from 13.9 ± 7.5 SD of *T*_0_ to 6.5 ± 5.9 SD of *T*_1_; mean difference 7.30; *p* < 0.001) (Figure 1), while for group B, this difference was not statistically significant (from 15.25 ± 10.1 SD of *T*_0_ to 12.5 ± 7.7 SD of *T*_1_; mean difference 2.75; *p* > 0.05).

The GLMM showed that male subjects tend to have fewer mean of attacks than females regardless of the time considered (9.79 ± 0.7 SD for males vs 14.11 ± 0.5 SD of the standard subject; linear coefficient: −0.37 vs 2.6 of the standard subject; *p*=0.01).

In addition, the GLMM shows us how the response to therapy is strongly dependent on the duration of treatment; in fact, patients who received the drug for at least 12 weeks had a greater response than patients who took it for less time. The linear coefficient for patients receiving PEA in general is −0.04 (vs 2.64 of the standard subject; *p*=0.8), while for patients treated at least 12 weeks, it is −0.6 (vs 2.65 of the standard subject; *p* < 0.01).

**Figure 2:**
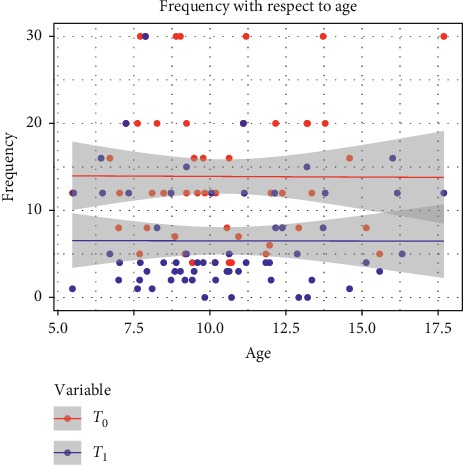
Result of logistic regression for correlation between age and response to treatment.

We did not find any significant correlation between age and the frequency of migraine attacks (*p* > 0.05) (Figure 2).

### 3.2. Effects on Intensity of the Attacks

**Figure 3:**
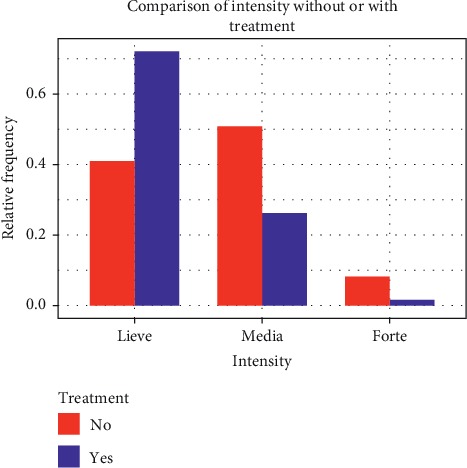
Comparison of intensity of migraine attacks before and after 12 weeks of treatment with umPEA.

We observed a reduction in intensity of the attacks from *T*_0_ to *T*_1_ in all the three groups but with significant differences only for the total population (from 1.71 ± 0.6 of *T*_0_ to 1.36 ± 0.5 of *T*_1_; *p* < 0.001) and group A (from 1.67 ± 0.6 of *T*_0_ to 1.31 ± 0.5 of *T*_1_; *p* < 0.001), while for group B, the difference was not significant (from 2.0 ± 0.7 of *T*_0_ to 1.75 ± 0.7 of *T*_1_; *p* > 0.05). For group A, the percentage of patients with severe attacks decreased after treatment (from 8.2% to 1.6%; *p* < 0.05) (Figure 3).

The CLMM showed that male subjects tend to have less intense attacks than females (linear coefficient: −2.21 vs the standard subject; *p* < 0.01). Regarding the effect of duration of treatment, we found that the linear coefficient for patients receiving PEA in general is −1.3 (*p*=0.2), while for patients treated at least 12 weeks, it is −2.4 (*p* < 0.01).

### 3.3. Effects on the Number of Medications Taken for the Attack

**Figure 4:**
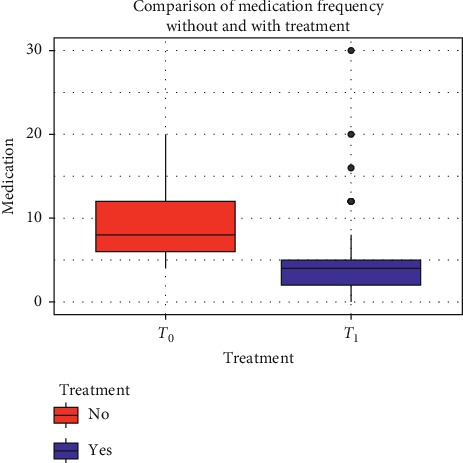
Comparison of monthly assumption of drugs for the attack before and after 12 weeks of treatment with umPEA.

The monthly assumption of drugs for the attack reduced from 9.2 ± 4.3 to 5.68 ± 5.5 (*p* < 0.001) for general population and from 9.5 ± 4.4 to 4.9 ± 2.5 (*p* < 0.001) (Figure 4), while for group B, it decreased from 11.13 ± 7.5 to 10.5 ± 7.2 (*p* > 0.05).

The GLMM showed no significant differences in male and female for numbers of drug intake (8.28 ± 4.4 SD for males vs 9.5 ± 4.4 SD of the standard subject; linear coefficient: −0.21 vs 2.3 of the standard subject; *p*=0.1).

The only parameter correlated with a reduction in the intake of the drugs for the attacks that is identified by the GLMM is the duration of treatment. The linear coefficient for patients receiving PEA regardless of the duration of therapy is −0.17 (vs 2.34 of the standard subject; *p* > 0.05), while for patients treated at least 12 weeks, it is −0.76 (vs 2.34 of the standard subject; *p* < 0.01).

### 3.4. Dosage and Tolerability

We found that patients who had at least a 50% decrease in attack frequency after treatment had received an average drug dose of 40 mg/kd/day of PEA.

Regarding safety, only one patient developed side effects and consisted of nausea and floating.

## 4. Discussion

Our study showed that treatment of pediatric migrainous patients with umPEA is well tolerated and improves the frequency, intensity, and duration of the attacks. These effects are generally observed for a duration of treatment not less than 12 weeks and at an average dosage of 40 mg/kg/day (maximum 600 mg/day).

Migraine is a complex disease in which different biochemical and neurophysiological abnormalities have been described. Specific neuronal, glial, and vascular signaling pathways involved in pathogenesis of migraine may represent distinct targets for acute and preventive migraine therapies [19].

The treatment of migraine in pediatric age encounters numerous obstacles considering that traditional medicines do not have the same evidence of efficacy as in adults, and the new drugs against CGRP have not been systematically tested in this age group. Furthermore, the high response to placebo raises doubt about the actual need to treat migraine children with drugs that may have adverse effects [20]. This is why the choice falls very often on nutraceuticals whose efficacy data however remain conflicting [8].

### 4.1. PEA in Migraine Prophylactic Treatment

To date, only one study has been conducted to evaluate the role of PEA in migraine management in adults [17]. The authors demonstrated that umPEA administration to patients with MA (1,200 mg/day for up 90 days) treated with common NSAIDs induced a significant pain relief irrespective to age or gender. These effects were evident at 60 days after the beginning of umPEA treatment and lasted throughout the study. These results are in agreement with the previous reports showing the antinociceptive action of umPEA in both preclinical models of neuropathic pain and with clinical trials performed in a variety of pain states [21].

In line with the study of Chirchiglia et al., our pilot study showed that umPEA at low doses (600 mg/kg/day) for short period (three months) is effective in reducing migraine attacks frequency and intensity in pediatric patients [17]. Our study also confirms the safety of treatment with umPEA. Indeed, no severe adverse drug reactions or interactions were recorded during the study highlighting an optimal umPEA pharmacological profile, and the adherence with the umPEA regimen was good.

Regarding the choice of the dose of umPEA to be administered, we have referred to other studies in pediatric and adult populations. In other pediatric diseases, such as acute respiratory infections, the dosage of 50 mg/kg (maximum ∼800 mg /day), for the age groups between 1 and 6 years and 11 and 16 years, was found to be safe and effective [22, 23]. In addition, the only study of umPEA in migraine used the doses of 600 mg/kg/day [17].

### 4.2. Possible Mechanisms of Action of PEA

PEA is an endogenous fatty acid amide signaling molecule synthesized “on demand” in response to tissue injury/stress, as part of a mechanism to restore/maintain homeostasis with anti-inflammatory, pain-relieving, and neuroprotective actions [24–27]. This view is supported by studies showing that PEA levels change in settings of tissue injury, especially in situations associated with inflammatory and neurodegenerative processes [27–30]. The anti-inflammatory effects of PEA seem to be mainly related to its ability to modulate mast cell (MC) activation and degranulation, and this action is also known as the ALIA (autacoid local inflammation antagonism) mechanism [31, 32]. In fact, MCs as well as glia possess endogenous homeostatic mechanisms that can be upregulated because of tissue damage or stimulation of inflammatory responses. Such molecules include the N-acylethanolamines, whose principal family members are the endocannabinoid N-arachidonoylethanolamine (anandamide), and its congeners N-stearoylethanolamine, N-oleoylethanolamine, and PEA. In particular, PEA is produced and hydrolyzed by microglia in response to stress inflammatory events and it downmodulates MC activation [25].

After the first description of the ALIA mechanism by Rita Levi Montalcini [33], other direct and indirect mechanisms explaining the PEA anti-inflammatory effects have been hypothesized. Though synergistic interactions involve several mechanisms, PEA can produce its important therapeutic effects, in both the central and peripheral nervous systems [12]. The direct targets of the PEA are two receptors: the PPAR-*α* [34] and the orphan GPR55 (GPR55) [35]. PEA has an agonist activity on the PPAR-*α* receptor that is a gene expression factor that promotes the expression of genes with anti-inflammatory activity [36]. GPR55 shows a low homology with cannabinoid receptors CB1 and CB2. It has been reported that GPR55 uses a variety of downstream signing events with regulatory effects of neuroinflammation [31]. The indirect effects of PEA are on the CB1 and CB2 receptors, the fatty acid amide hydrolase (FAAH) factor, and the transient receptor potential vanilloid 1 (TRPV1) receptor channel. Through the inhibition of the FAAH expression, PEA may increase the endogenous levels of anandamide (AEA) which directly activate CB2 or CB1 receptors and TRPV1 channels (entourage effect) [12, 37]. The final effect is an activation of CB1 and CB receptor and desensitization TRPV1 channel [38]. CB1 is expressed in brain tissue and regulates neuronal transmission [27]. CB2 is expressed on mast cells, activated microglia, controls inflammation, and nociceptive signals [26]. TRPV1 channel receptors are widely expressed in small sensory C and A delta fiber. They have also been observed in the central nervous system and in physiological membranes of several tissues. TRPV1 have also been observed in many organs, and their increased expression contributes to development and perception of the somatic and visceral pain. The process of TRPV1 inactivation, also known as “desensitization,” contributes to the analgesic and anti-inflammatory actions of TRPV1 agonists [39].

PEA is produced and hydrolyzed by microglia [40], and through several abovementioned mechanisms, it inhibits mast cell activation [31, 41]. Although PEA has been studied in several inflammatory diseases, there are very few data on its efficacy in migraine. The recent observation of mast cell involvement in some mechanism of migraine could explain the efficacy of this molecule for the treatment of this condition [42].

The effect of MCs resident in the meninges can play a critical role in the development of inflammation in many diseases, including migraine [43]. Since several original studies by Theoharides et al. [44], the role of meningeal mast cells as triggers of migraine attacks was further explored by others, showing the pronociceptive role of mast cell derived from proinflammatory cytokines and chemokines [42, 43, 45, 46]. MCs are densely present in meningeal tissues, located adjacent to both nerves and vessels [43, 44]. The contact between MCs and nerve endings forms a neuroimmune synapse where active substances released by MCs can activate neighboring nociceptive fibers, and compounds released from active fibers, in turn, can degranulate MCs [47]. Degranulation of dural MCs can strongly activate meningeal nerve fibers [48]. In fact, activated MCs release preformed mediators including histamine, heparin, proteases (tryptase and chimase), hydrolases, cathepsin, carboxypeptidases, and peroxidase, and they also generate proinflammatory cytokines and chemokines. In addition, activated macrophages, microglia, and MCs in the CNS release proinflammatory cytokines which provoke an increase of arachidonic acid product levels and lead to migraine and other neurological manifestations including fatigue, nausea, headaches, and brain fog [46].

### 4.3. Limitations of the Study

We must underline that this study has some limitations represented. First, we did not use a placebo-controlled design. Given the huge effect of placebo demonstrated in the prophylaxis of pediatric migraine [49], the lack of a control group treated with placebo prevents us to reach a definitive conclusion about umPEA efficacy. Second, although the umPEA was effective in most our patients, the studied sample was relatively small. Third, our follow-up was brief, thus preventing us to have data about the duration of the umPEA effect. In spite of all these limitations, our pilot study represents the first investigation about the possible efficacy of the umPEA in pediatric migraine. Of course, the present promising results need to be confirmed in a larger population and after comparison with placebo.

## 5. Conclusion

Although PEA is not reported in guidelines of migraine treatment in pediatric age, in our study, the administration of umPEA to pediatric patients with migraine induced a significant pain relief and reduction of the number of migraine attacks. No major adverse drug reactions or interactions were recorded during the study. These data, suggesting an optimal pharmacological profile, lead us to conclude that PEA should be considered for prophylactic treatment of migraine in children.

## Data Availability

The clinical data used to support the findings of this study are included within the article.
